# Allopurinol-Induced Drug Reaction with Eosinophilia and Systemic Symptoms Syndrome: A Cause of Acalculous Cholecystitis?

**DOI:** 10.7759/cureus.1569

**Published:** 2017-08-16

**Authors:** Husnain Waseem, Faisal Inayat, Madina Abduraimova, Stephan Kamholz

**Affiliations:** 1 Internal Medicine, Maimonides Medical Center; 2 Department of Medicine, Allama Iqbal Medical College, Lahore, Pakistan; 3 Chair, Department of Medicine, Maimonides Medical Center

**Keywords:** dress syndrome, allopurinol, hypersensitivity, acalculous cholecystitis, new entity

## Abstract

Acalculous cholecystitis (AC) is an inflammation of the gallbladder in the absence of gallstones. There are many risk factors associated with AC. However, this report implicates allopurinol as an inciting agent for a severe systemic drug reaction, i.e., the drug reaction with eosinophilia and systemic symptoms (DRESS) syndrome complicated by AC. We report a Chinese woman who presented on two occasions with a diffuse maculopapular rash, elevated liver enzymes, and upper abdominal pain attributable to acute AC, the second episode of which developed after the reintroduction of allopurinol treatment for gout. The AC complicated the DRESS syndrome during the course of her hospitalization.

## Introduction

Acute acalculous cholecystitis (AC) is defined as a severe gallbladder inflammation without gall-stone-related cystic duct obstruction. It causes 2%-15% of all episodes of acute cholecystitis [1]. The usual predisposing factors associated with the development of AC include recent surgery, burns, trauma, medical illnesses, such as diabetes mellitus, vasculitides such as systemic lupus erythematosus (SLE) or polyarteritis nodosa, congestive heart failure, and cystic artery occlusion [2].

## Case presentation

A 71-year-old Chinese woman presented to the emergency department (ED) with a generalized body rash of one-day duration. The rash started one day after the patient reinitiated allopurinol treatment for the spontaneous onset of bilateral ankle pain associated with redness and swelling. The patient reported two days of intermittent fever ranging from 100.4 °F to 101.4 °F. She denied the history of recent travel, tick bite, pain in other joints, and myalgias. Past medical history included hypertension, dyslipidemia, diabetes mellitus, and recently diagnosed gout. She was recently diagnosed with gout and allopurinol therapy was initially started four weeks prior to her current presentation.

The patient revealed that she was hospitalized with a similar rash, high-grade fever, and upper abdominal pain two weeks previously. The patient took allopurinol for one week and stopped it after her ankle pain resolved. Physical examination revealed right upper quadrant tenderness, and palpation elicited Murphy's sign (associated with acute cholecystitis). Skin examination revealed a petechial, non-blanching rash on all extremities. A poorly defined, diffuse, and nonscaling erythematous maculopapular rash covered the trunk. The vital signs were—heart rate: 135 beats/min, respiratory rate: 25/min, and temperature: 102.9 °F. In the ED, she developed hypotension of 90/60 mmHg.

A computed tomography (CT) scan of the abdomen demonstrated nonspecific pericholecystic fluid and periportal edema. Abdominal ultrasonography revealed a thickened gallbladder wall measuring 7 mm and the presence of pericholecystic fluid. There were no visible stones. No intra or extrahepatic bile duct dilatation was noted. The proximal common bile duct (CBD) measured 3.5 mm. The initial laboratory evaluation revealed white cell count (WBC): 2.3 x 10^3^/µL (4-11 x 10^3^/µL), platelet count: 158 x 10^3^/µL (150-450 x 10^3^/µL), lactate: 3.9 mEq/L (0.5-1.3 mEq/L), blood urea nitrogen (BUN): 12 mg/dL (10-26 mg/dl), creatinine: 1.4 mg/dL (0.6-1.3 mg/dL), total bilirubin: 2.2 mg/dL (0.2-1 mg/dl), direct bilirubin: 0.7 mg/dL (0 to 0.3 mg/dL), aspartate aminotransferase (AST): 71 IU/L (7-40 IU/L), alanine aminotransferase (ALT): 56 IU/L (0-40 IU/L), alkaline phosphatase (ALP): 100 IU/L (70-230 IU/L) , and serum lipase: 45 IU/L (0-140 IU/L). Therein, the patient was diagnosed with acute AC. 

Intravenous (IV) vancomycin (1500 mg), cefepime (2 gm), and methylprednisolone (60 mg) were administered in addition to aggressive fluid resuscitation. Percutaneous cholecystostomy and drain placement were accomplished without complications. Blood and urine cultures were persistently negative. Resolution of the rash occurred on Day 3 of hospitalization and, subsequently, the corticosteroid therapy was discontinued. On the fourth hospital day, laboratory data included WBC: 12.9 x 10^3^/uL, platelets: 52 x 10^3^/uL, total bilirubin: 2.4 mg/dL, direct bilirubin: 1.5 mg/dL, lactate: 1.3 mEq/L, and creatinine: 0.8 mg/dL. On the fourth, fifth, and sixth hospital days, eosinophil percentages were 17%, 14.2%, and 9.5%, respectively. The European Registry of Severe Cutaneous Adverse Reactions (RegiSCAR) score was four, suggesting the possibility of the drug reaction with eosinophilia and systemic symptoms (DRESS) syndrome. Antimicrobial therapy was changed to IV ceftriaxone and IV metronidazole on the fourth day. The patient showed significant improvement in the rash and started tolerating oral feeding. She was discharged from the hospital on Day 8. At the time of discharge, the etiology of acute AC had not been determined with certainty. Allopurinol was not discontinued at the time of discharge.

**Figure 1:**
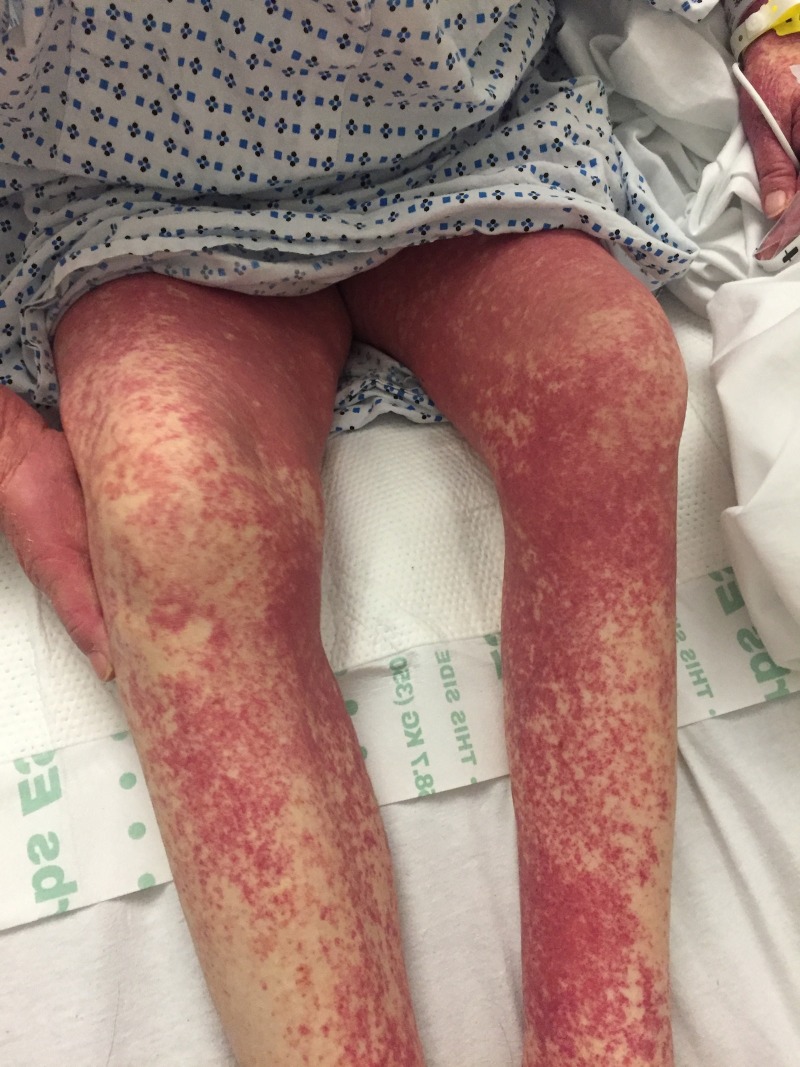
Rash on lower extremities at the time of presentation

**Figure 2:**
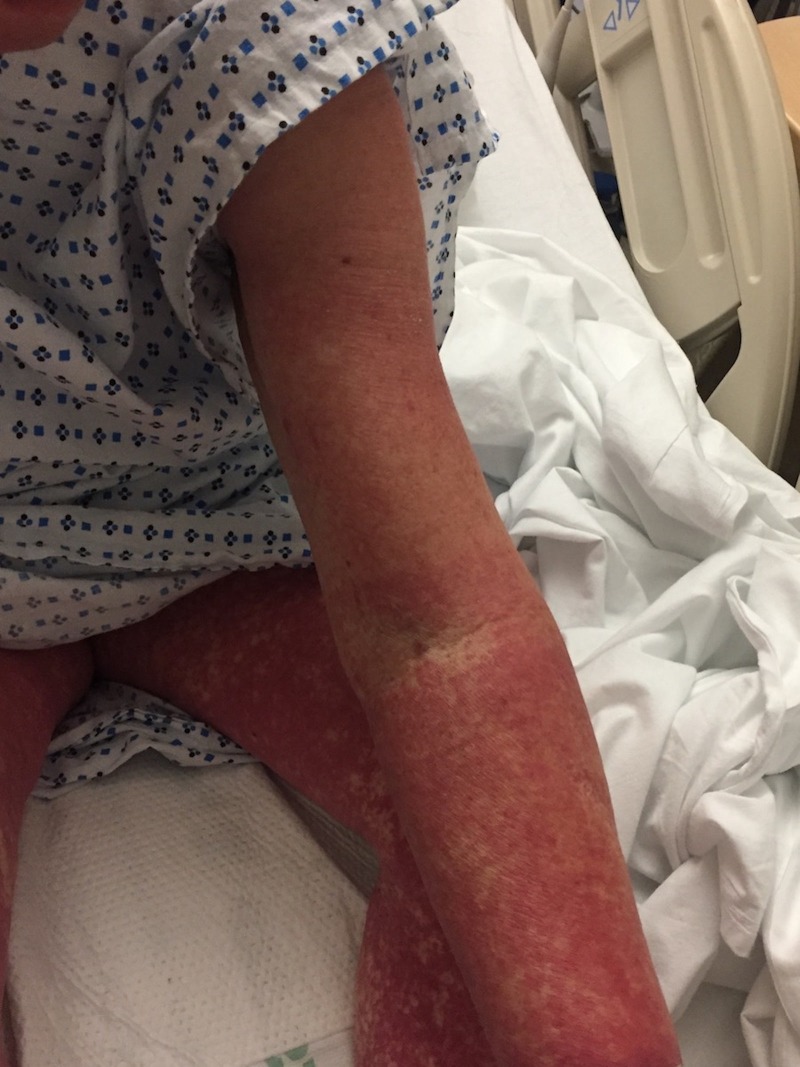
Rash on upper extremity at the time of admission

Five days after hospital discharge, the patient developed left-sided ankle pain and again took one dose of 300 mg allopurinol. Within 24 hours, a diffuse erythematous rash all over her body recurred, and she returned to the emergency department. Examination revealed temperature: 103.7 °F, heart rate: 117 beats/minute, respiratory rate: 20/minute, and blood pressure: 161/61 mmHg. A morbilliform rash was present over the pubic symphysis. A petechial rash was noted on both legs (Figure 1) as well as on the upper extremities (Figure 2).

**Figure 3:**
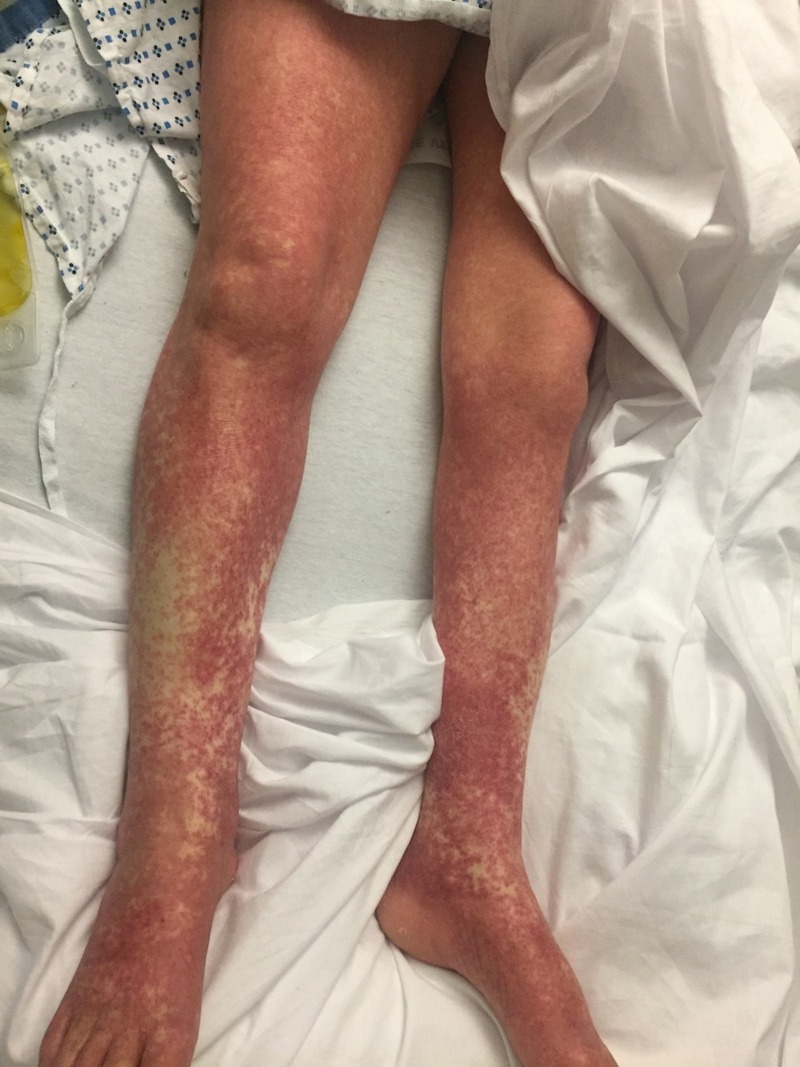
Resolving erythematous lesions on lower limbs at the time of discharge

**Figure 4:**
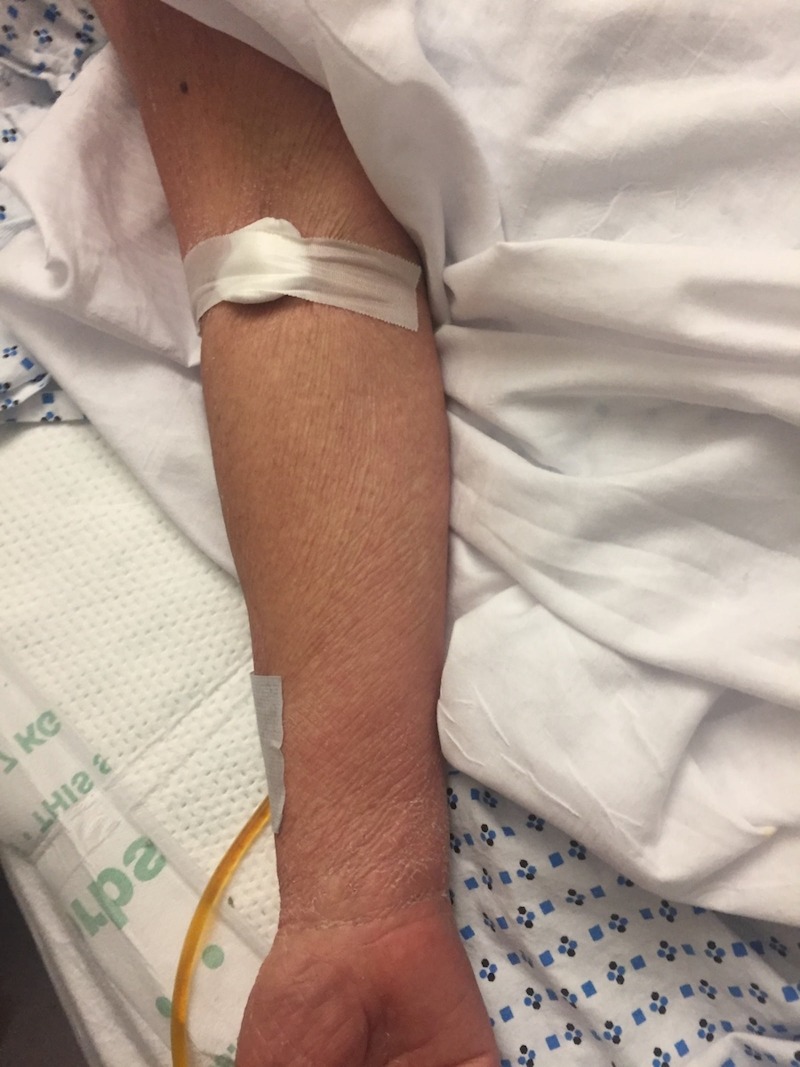
Resolution of rash on upper limb at discharge

The oral mucosa was uninvolved. The left ankle was warm and tender to touch and a mild effusion was present. Laboratory data included WBC: 9.4 x 103 /uL (4-11x103/μL), eosinophil: 31% (1%-3%), neutrophils: 72.5% (57-67%), C-reactive protein: (CRP) 9.100 mg/dL (1-3 mg/dL), erythrocyte sedimentation rate (ESR): 90 mm/hr (0-20mm/hr), lipase: 170 U/L (0-140 U/L), B-type natriuretic peptide (BNP): 177 pg/mL (0-100pg/mL), and uric acid: 5.9 mg/dL (2.1-6.3 mg/dl). Radiographic imaging of the chest, abdomen, and pelvis did not reveal any abnormalities. The diagnosis of "possible" DRESS syndrome due to allopurinol hypersensitivity was made on a RegiSCAR score of three. Intravenous methylprednisolone (40 mg) was administered twice daily for six to seven days. Significant resolution of the rash occurred during treatment. The upper left arm was almost cleared of the rash (Figure 3). Residual post-inflammatory changes were noted on both legs (Figure 4). Corticosteroid therapy was discontinued. The patient was educated about her allergy to allopurinol and about not taking this medication in the future.

## Discussion

Allopurinol hypersensitivity is a rare adverse reaction that is characterized by cutaneous manifestations, ranging from benign maculopapular eruptions to serious disorders such as Stevens–Johnson syndrome and toxic epidermal necrolysis. The reaction may be accompanied by fever, hepatic dysfunction, renal impairment, eosinophilia, and leukocytosis [3]. One of the clearest risk factors for developing allopurinol toxicity is Han-Chinese ethnicity, especially those individuals carrying the human leukocyte antigen (HLA)-B 5801 alleles [3-5]. Although HLA phenotyping was not available for this patient, she was of Han-Chinese descent.

The DRESS syndrome was first described in 1996. Fever, lymphadenopathy, rash, and internal organ involvement with marked eosinophilia are common manifestations of this disease. The most frequently involved organ is the liver, followed by the kidney and lungs. Allopurinol was found to be the culprit drug in 19% of the cases in a literature review of 172 cases [6]. The pathophysiology of the DRESS syndrome remains unclear, but a defect in the detoxification of the causative drug, immunological imbalance, and infections such as human herpes virus type 6 (HHV 6) are previously suggested [7]. The overall mortality in DRESS is about 10% and occurs in patients with severe multiorgan involvement.

The absence of gallstones on abdominal imaging and studies performed at the time of initial admission mitigated against acute gallstone-induced cholecystitis. The patient did not have common risk factors for AC such as trauma, burns, recent surgery, and sepsis, as blood and urine cultures were persistently negative. Because the association of the symptoms and signs attributable to allopurinol were not noted during the initial hospitalization, the drug was not discontinued. We hypothesize that the systemic hypersensitivity reaction, systemic inflammatory response syndrome (SIRS), and DRESS syndrome secondary to allopurinol toxicity could have been the cause of this patient's AC.

It is likely that our patient developed AC during the first hospital admission due to a hypersensitivity reaction caused by allopurinol. There are several reasons to support this contention: (a) The patient was recently started on allopurinol four weeks prior to the first episode of rash and right upper quadrant abdominal pain. One report suggested that the median time to the onset of allopurinol hypersensitivity was 30 days [4]. (b) This patient manifested a similar rash on both occasions. (c) No alternative etiology of AC was identified on imaging studies, and the blood cultures were persistently negative. (d) The patient again developed a similar rash one day after she resumed allopurinol because she was not instructed to stop the medication at the time of her discharge.

The pathogenesis of AC involves endothelial injury, gallbladder ischemia, and stasis, leading to a concentration of bile salts, gallbladder distension, and, eventually, necrosis of the gallbladder tissue. Once AC is established, secondary infection with enteric pathogens, including Escherichia coli, Enterococcus faecalis, Klebsiella spp, Pseudomonas spp., Proteus spp., and Bacteroides spp. is common. Gallbladder perforation occurs in severe cases. The presence of a characteristic rash, the recent use of allopurinol, and the co-development of AC allows us to speculate that AC was related to the allopurinol-induced hypersensitivity reaction. Endothelial injury, bladder ischemia, a release of inflammatory cytokines, such as Interleukin (IL)-1, IL-6, and tumor necrosis factor (TNF), and stasis secondary to SIRS and the DRESS syndrome could have caused the insults to the gallbladder.

The treatment of AC includes the initiation of antibiotics after obtaining blood cultures. Cholecystostomy is often preferred over cholecystectomy as a less invasive option in critically ill patients. Delay in treatment can result in gallbladder perforation and gangrene and mortality rates may be as high as 70% in such patients. While the placement of a cholecystostomy tube is common, cholecystectomy should be performed if there are findings suggesting gallbladder necrosis, emphysematous cholecystitis, or perforation. These findings were absent in our patient.

## Conclusions

The present report highlights the importance of thorough medication history in patients presenting with symptoms of AC, especially in those with no known cause of gallbladder inflammation. A systemic hypersensitivity reaction associated with drug allergy, such as the DRESS syndrome, may cause AC. Therefore, clinicians should maintain a high index of suspicion, especially in patients taking one of the drugs commonly known to cause severe systemic hypersensitivity reactions such as allopurinol.
